# Token Economy–Based Hospital Bed Allocation to Mitigate Information Asymmetry: Proof-of-Concept Study Through Simulation Implementation

**DOI:** 10.2196/28877

**Published:** 2022-03-04

**Authors:** Shusuke Hiragi, Jun Hatanaka, Osamu Sugiyama, Kenichi Saito, Masayuki Nambu, Tomohiro Kuroda

**Affiliations:** 1 Division of Medical Informatics and Administration Planning Kyoto University Hospital Kyoto Japan; 2 Graduate School of Informatics Kyoto University Kyoto Japan; 3 Division of Health Science Tazuke Kofukai Medical Research Institute Osaka Japan; 4 Department of Real World Data Research and Development Graduate School of Medicine Kyoto University Kyoto Japan; 5 Preemptive Medicine & Lifestyle-Related Disease Research Center Kyoto University Hospital Kyoto Japan

**Keywords:** hospital administration, resource allocation, token economy, bed occupancy, hospital management, simulation, decision-making, organization

## Abstract

**Background:**

Hospital bed management is an important resource allocation task in hospital management, but currently, it is a challenging task. However, acquiring an optimal solution is also difficult because intraorganizational information asymmetry exists. Signaling, as defined in the fields of economics, can be used to mitigate this problem.

**Objective:**

We aimed to develop an assignment process that is based on a token economy as signaling intermediary.

**Methods:**

We implemented a game-like simulation, representing token economy–based bed assignments, in which 3 players act as ward managers of 3 inpatient wards (1 each). As a preliminary evaluation, we recruited 9 nurse managers to play and then participate in a survey about qualitative perceptions for current and proposed methods (7-point Likert scale). We also asked them about preferred rewards for collected tokens. In addition, we quantitatively recorded participant pricing behavior.

**Results:**

Participants scored the token economy–method positively in staff satisfaction (3.89 points vs 2.67 points) and patient safety (4.38 points vs 3.50 points) compared to the current method, but they scored the proposed method negatively for managerial rivalry, staff employee development, and benefit for patients. The majority of participants (7 out of 9) listed human resources as the preferred reward for tokens. There were slight associations between workload information and pricing.

**Conclusions:**

Survey results indicate that the proposed method can improve staff satisfaction and patient safety by increasing the decision-making autonomy of staff but may also increase managerial rivalry, as expected from existing criticism for decentralized decision-making. Participant behavior indicated that token-based pricing can act as a signaling intermediary. Given responses related to rewards, a token system that is designed to incorporate human resource allocation is a promising method. Based on aforementioned discussion, we concluded that a token economy–based bed allocation system has the potential to be an optimal method by mitigating information asymmetry.

## Introduction

Hospital bed management is an important resource allocation task for better patient care and sustainable hospital management [1]. In particular, efficient bed utilization is a key driver for hospital revenue [2] and health care system management [3,4]. The management of hospital finances has become difficult in Japan [5,6] and worldwide [7-9]; thus, to cope, effective resource management is essential.

Poor bed management wastes time, resulting in longer wait times for patients, the reduction of employee (especially ward nurses) satisfaction [10], and adverse effects on patient safety [11,12].

The current bed control process begins when physicians admit patients to the hospital and asked bed allocation managers to find adequate beds [13]. Allocation managers search the wards to find the best fit for the patient. Then, either the *negotiation-based method*, wherein allocation managers negotiate with frontline ward managers, who sometimes decline the request, to form consensus and make a final decision [14], or the *command-based method*, wherein allocation managers have the authority to force wards to accept the request [15], emerges. The negotiation-based method takes more time [14], while the command-based method hinders employee satisfaction by involuntarily increasing the patient-to-nurse ratio, which has been reported to increase staff dissatisfaction [16]. In fact, these methods are not discrete but form a continuum, as procedures, in reality, both have negotiation-based as well as command-based aspects.

Conflict is defined as the struggle that arises when the goal-directed behavior of a person or group blocks that of another [17]. Both bed allocation methods include a systemic structure that can cause intraorganizational conflict between allocation managers and frontline ward workers. Previous reports mentioned that mediation of information asymmetry is an effective form of conflict management [18], and often, information asymmetry is observed in real practice between allocation managers, who have broad but superficial views of the bed occupancy situation for the whole hospital, and managers of individual wards, who tend to only see their situation but perfectly know their capacity.

Previous studies [19,20] in operational research have tried to solve the bed assignment problem with mathematical modeling, which is based on the assumption that computers can calculate optimal solutions for command-based bed allocation to satisfy any stakeholder requirements; despite this, no real-world solutions are in wide use, because of the implementation difficulties arising from inputting data from a variety of sources and convincing users to accept computed suggestions [19]. This situation indicates that collecting sufficient information is difficult, which can lead to information asymmetry, resulting in intraorganizational conflict.

In the field of economics and management, *signaling*, whereby one party credibly conveys some information about itself to another [21], is a solution for information asymmetry [22]. However, requiring too much information from frontline staff is not feasible in practice; therefore, simple information to be expressed is needed. Herein, we propose the implementation of a token economy–based operation for bed allocation, in which the allocation process is regarded as a virtual currency transaction. Token economy was originally developed for behavior modification in psychology [23] and education [24] and is now also used by businesses [25] that intend to manage user behavior. We hypothesize that this system has the advantages of both negotiation-based and command-based allocation systems.

We developed a token economy–based system for bed allocation and conducted a preliminary evaluation of the system with a game-like simulation followed by a brief survey about its effectiveness.

## Methods

### Token Economy–Based Bed Assignment System

#### Overview

**Figure 1:**
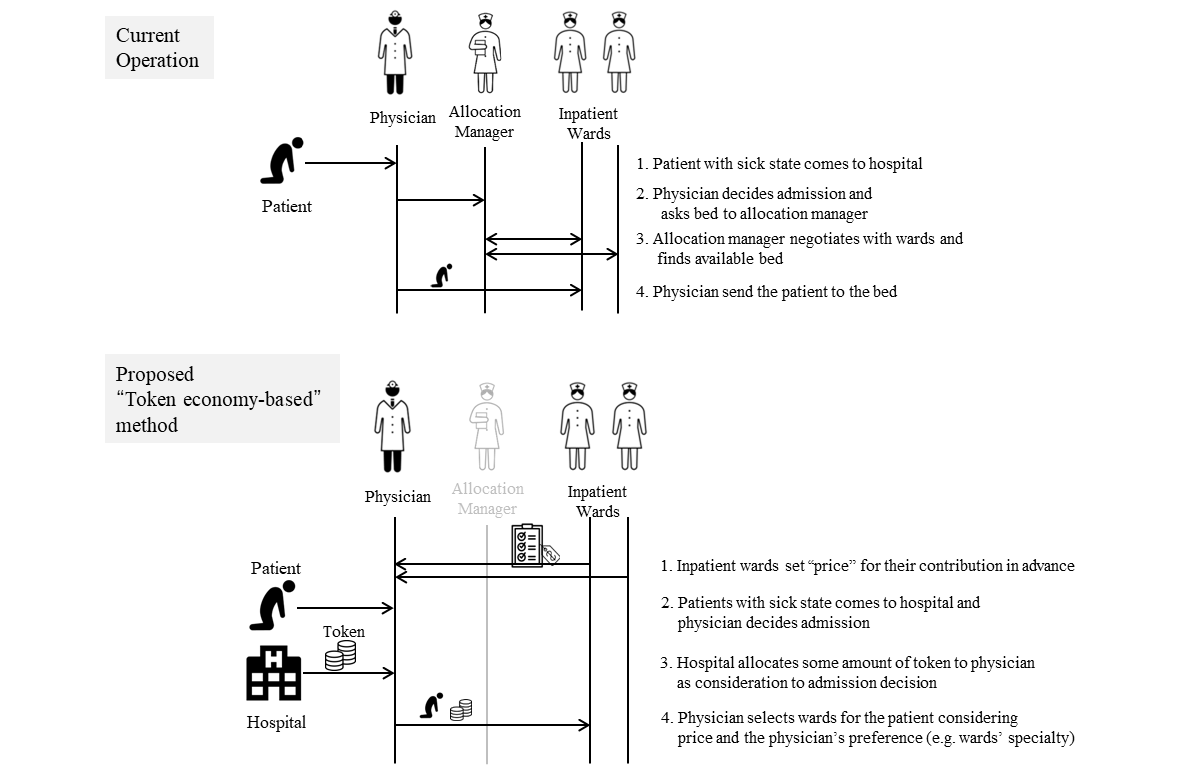
Operation flowchart of current (upper) and proposed (lower) bed allocation methods.

The current bed assignment process begins when physicians decide to admit a patient to the hospital and the allocation manager negotiates with ward staff to find an available bed (Figure 1).

However, we propose that the bed allocation process can be considered as transactions of nursing contributions between physicians and nursing staff. Each ward in the hospital is asked to set a *price* (ie, define the value for their nursing service) using a virtual currency (ie, token) in advance. When physicians admit patients, the hospital allocates a certain number of tokens to the physicians as consideration for admission. Then, using *prices* and their preferences, such as ward specialty, physicians decide where patients will be assigned. Once the deal is finalized, the bed allocation process is completed (Figure 1). In this process, allocation managers do not have a role. By repeating this procedure, each stakeholder can accumulate tokens, to be exchanged for something that has value to them, and ward *price* setting acts as a signaling of availability, which removes information asymmetry to result in optimal bed allocation.

#### Implementation of Simulation System

To evaluate the token economy–based bed allocation system, we developed a game-like simulation. We developed a web-based simulation for nurse managers (who represent the nursing staff on the wards). The behavior of physicians was emulated by computer agents.

**Figure 2:**
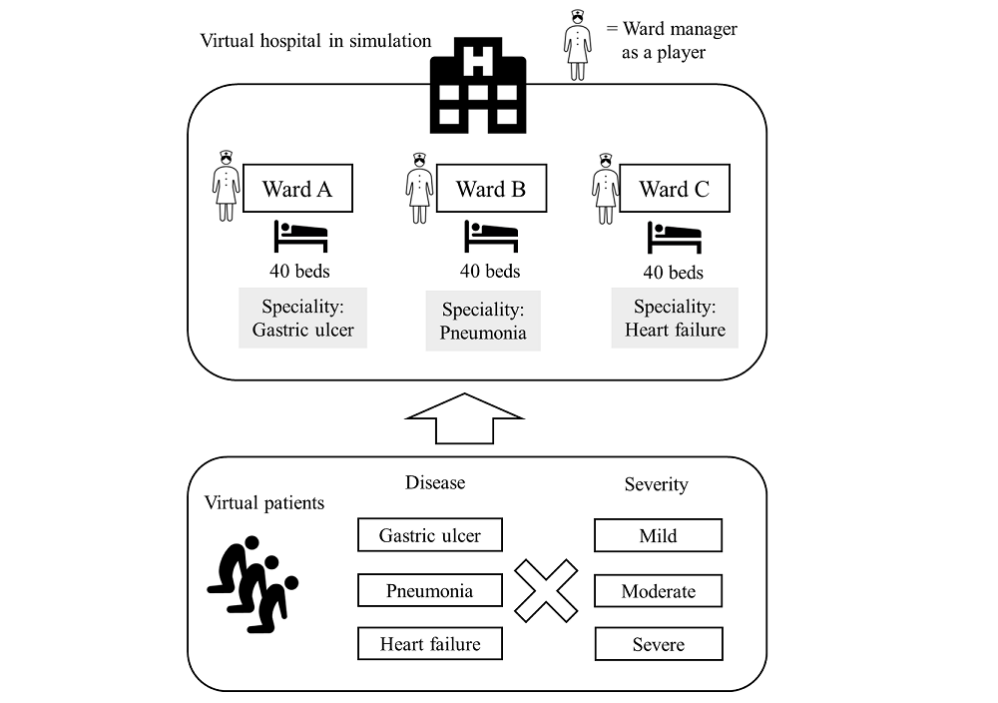
Game scenario: the hospital consisted of 3 wards, with 40 beds each, and patients with 3 diseases with 3 levels of severity.

The scenario consisted of a small hospital with 3 wards, each having 40 beds. For simplicity, we used the assumption that there are 3 illnesses (ie, gastric ulcer, pneumonia, and heart failure) with 3 severities (ie, mild, moderate, and severe). To represent the real-world allocation of specialties, we also defined the assumption that each virtual ward was used to manage a single type of illness: ward A, gastric ulcer; ward B, pneumonia; and ward C, heart failure (Figure 2).

Each virtual ward had a *workload* parameter, that reflected nursing staff workloads. This information was only seen by the relevant ward, thus representing information asymmetry. The workload increased in response to bed occupancy, weighted by patient illness and severity: *workload* increased by 1.0, 1.25, and 1.5 points when the ward received a patient with mild, moderate, and severe, respectively, corresponding illness; however, when the patient’s illness was not aligned with the ward’s specialty, *workload* increased by an additional 50%—1.5, 1.8, and 2.25 points for mild, moderate, and severe illnesses, respectively, not corresponding to that of the ward specialty (Table S1 in Multimedia Appendix 1).

#### User Interface

**Figure 3:**
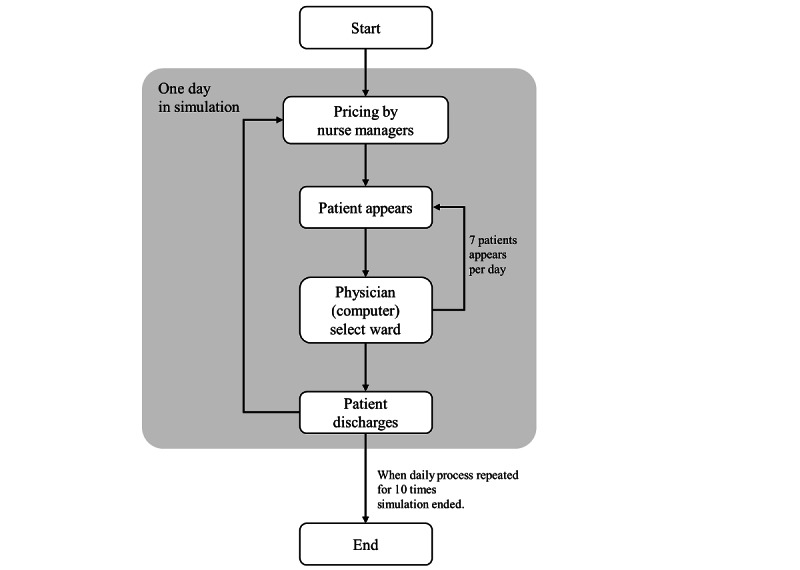
Flowchart illustrating the game. The gray area indicates 1 day in the simulation, with the game consisted of 10 virtual days.

The simulation consisted of day-level repetition (Figure 3). At the beginning of a day, nurse managers (players) were asked to set a *price* for each illness and severity. They could see the current bed occupancy and staff workload of their ward, but they could only see the number of patients admitted to other wards (Figure S1 in Multimedia Appendix 1). Players were then asked asked to set *prices* for 9 patterns of patients (Figure S2 in Multimedia Appendix 1). Each day, 7 patients appeared, with random illnesses and severities (Figure S3 in Multimedia Appendix 1) and were allocated to the ward selected by physicians—a computer agent programmed to select the ward that proposed the lowest *price* unless the ward specializing in the patient’s illness was available, in which case, a certain additional *price* was acceptable. The additional rate was set as a uniform random number between 1.0 (no additional fee) and 2.0 for every admission decision. Patients were randomly discharged from wards at a rate equivalent to a mean of 14 days, so that vacant beds appeared in wards. We repeated the simulation 10 times, representing 10 real-world days. All simulations were implemented with Python (version 3.4.5) and Django (version 2.0.13).

### Evaluation

#### Participants

We recruited 9 nurse managers from a single university-affiliated hospital in Japan. We split them into 3 groups with 3 participants each. After receiving informed consent, we asked the nurse managers to participate in 2 sessions of the game-like simulation. During the first session, participants were told that the player who earned the most tokens would be the winner. For the second session, participants were instructed to assume they can receive the form of compensation they wish and that they are required to make decisions as nurse managers, by considering aspects such as staff satisfaction, patient safety, and equality, as they did in their daily practice.

**Figure 4:**
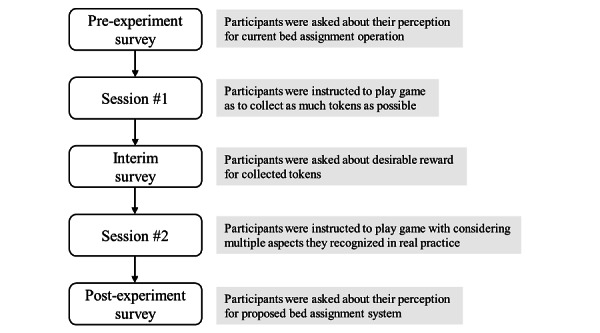
Evaluation flowchart.

We conducted 3 surveys with the participants: before, between, and after sessions (Figure 4). Using a similar question format about perceptions relating to manager satisfaction, staff satisfaction, benefit for patients, patient safety, timeliness of decision making, extent of managerial rivalry, extent of managerial control from hospital administrators, effect on revenues, employee development, consistency with organizational mission (which is equal to the whole hospital’s mission), and consistency with the goal of each ward as independent division (Table 1), the before-session survey asked participants about their perceptions of the current bed assignment practices, whereas the after-session survey asked participants about their perceptions of the token economy–based assignment method. Each response used a 7-point Likert scale (1, strongly disagree, to 7, strongly agree). We developed the questionnaire to examine the system’s effect on hospital management through resource allocation decision-making, given the advantages and disadvantages that have been identified by previous researchers [26], of decentralized decision-making in an organization. In addition, we considered Balanced scorecard, which is well-known organizational performance assessment tool [27]. In particular, questionnaire about employee development was set to evaluate this method could develop ward staffs’ managerial ability. We did not perform statistical testing, due to the limited number of participants.

For the interim survey, we asked participants about desired compensation regarding the token—“Please enumerate what you want to get as a consideration of tokens. List at least two, and at most five, items with giving priority.”

**Table 1:** Before- and after-session survey questions.

Topic	Current bed management practices	Proposed token economy–based system
Managers’ satisfaction	Are you satisfied with it?	Will you be satisfied with it?
Staff satisfaction	Do you think your staff is satisfied with it?	Do you think your staff will be satisfied with it?
Benefit for patients	Do you think it is beneficial for patients?	Do you think it will be beneficial for patients?
Patient safety	Do you think it has problems from the patient safety point of view?	Do you think it will have problems from the patient safety point of view?
Timeliness	Do you think it offers prompt decision-making?	Do you think it will offer prompt decision-making?
Managerial rivalry	Do you think it evokes managerial rivalry?	Do you think it will evoke managerial rivalry?
Control	Do you think managerial governance is maintained though it?	Do you think managerial governance will be maintained though it?
Revenue	Do you think it is optimized for hospital revenue?	Do you think it will be optimized for hospital revenue?
Employee development	Do you think it helps your staffs’ employee development?	Do you think it will help your staffs’ employee development?
Hospital mission	Do you think it is consistent with the hospital mission?	Do you think it will be consistent with the hospital mission?
Goal of divisions	Do you think it considers the goal of each ward?	Do you think it will consider the goal of each ward?

#### Analysis of Participants’ Price Setting

We recorded *price* list input by participants and *workload* fluctuations. We collected data from 6 sessions (3 groups, each playing 2 sessions). To evaluate this token economy–based method as a signaling mediator, we plotted the associations between *workload* and *price* setting.

#### Ethics Approval

These experiments were conducted with the approval of the Ethics Committee of Kyoto University Graduate School and Faculty of Medicine (R1972).

## Results

### Participants

Participants had more than 25 years (mean 31.1 years) of experience as nurses and more than 3 years (mean 11.6 years) as nurse managers at the time of experiment (Table 2).

**Table 2:** Demographic backgrounds of participants.

Characteristics			Value
**Gender (n=9), n (%)**			
	Male	1 (11)	
	Female	8 (89)	
**Experience (years), mean (range)**			
	Nurse	31.1 (26-34)	
	Nurse manager	11.6 (4-18)	

### Survey Results

**Figure 5:**
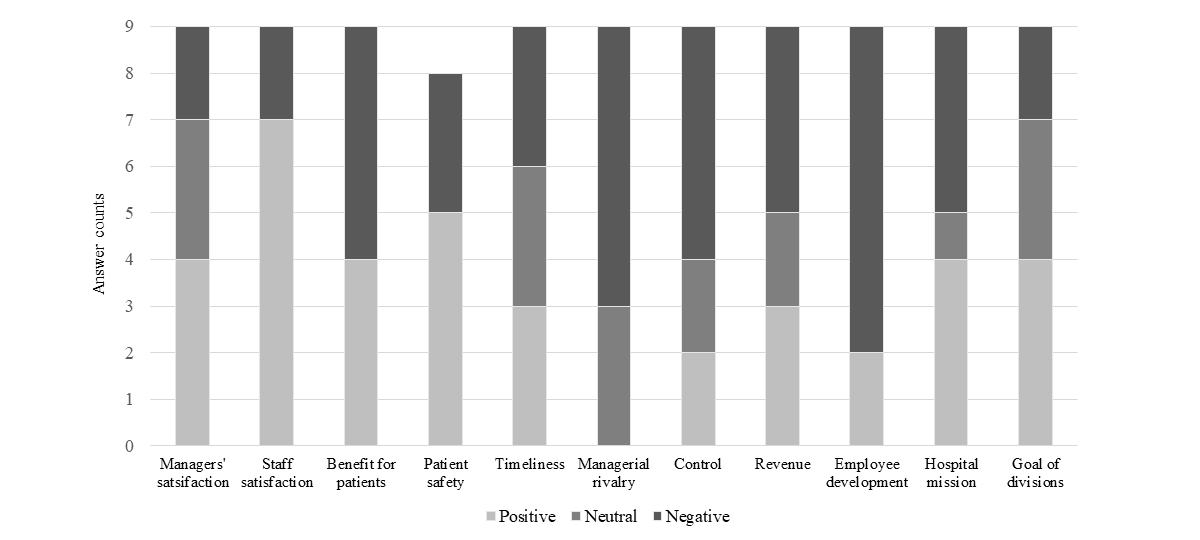
Differences between perceptions in current bed management practices and those for the proposed bed management method. Since a higher score for managerial rivalry indicates a negative response, we inverted the raw data. There was 1 missing response for patient safety.

Staff satisfaction for current operation practices and for the proposed method were at 2.67 and 3.89 points on average, respectively. Conversely, they scored benefit for patients in current practices and for the proposed method at 4.44 and 3.56 points, respectively. Participants gave favorable responses for the proposed method when compared with those for current practices for manager satisfaction, patient safety, timely decision-making, and consistency with goal of divisions; however, they gave unfavorable responses for the proposed method compared with those of current practices for the extent of managerial rivalry, the extent of control from hospital administration, employee development, and consistency with hospital mission (Table 3 and Figure 5).

Most participants (7 out of 9) listed additional human resources as preferred compensation for tokens. Financial compensation, such as salary increases or bonuses, ranked in the top 3 for some participants (4 out of 9). Only 1 participant did not list items related to human resources; this participant listed commendation from the hospital as the top priority (Table 4).

**Table 3:** Before- (current) and after-session (proposed) survey scores; items were rated on a 7-point Likert scale (1, strongly disagree, to 7, strongly agree).

	Current bed management practices, mean	Proposed token economy–based system, mean	Difference
Manager satisfaction	3.44	3.78	0.33
Staff satisfaction	2.67	3.89	1.22
Benefit for patients	4.44	3.56	−0.89
Patient safety^a^	3.50	4.38	0.88
Timeliness	3.67	4.00	0.33
Managerial rivalry^b^	2.78	3.67	0.89
Control	4.44	3.44	−1.00
Revenue	3.78	3.78	0.00
Employee development	4.11	3.44	−0.67
Hospital mission	4.56	4.11	−0.44
Goal of divisions	3.00	3.67	0.67

^a^There was a missing response; therefore, n=8 for this item.

^b^Only for this item, a higher score means a negative response.

**Table 4:** Participants’ response to the question asking the desirable rewards for collected token.

Participant	Priority				
	First	Second	Third	Fourth	Fifth
1	Human resources (nurses)	Travel fee for workshop	Travel fee for conference	Spare time or bonus	Improved work environment
2	Human resources (nurses)	Travel fee for conference	Improved work environment	Human resources (nurse assistants)	Equipment
3	Human resources (nurses)	Vacation	Bonus	Reduction of overtime	Support for mental condition
4	Human resources (nurses)	Bonus	Office supplies	Equipment	Human resources (doctors)
5	Allowance	Human resources (nurses)	Vacation	Spare time	Recreation fee
6	Human resources (nurse)	Human resources (nurse assistants)	Improved work environment	Education materials	—^a^
7	Human resources (nurses)	Salary	Human resources (doctors)	Improved work environment	—
8	Commendation	Equipment	Recreation fee	—	—
9	Human resources (nurses)	Spare time	Equipment	—	—

^a^Participants were asked to list between 2 and 5 items; therefore, some entries are blank.

### Workloads and Price Setting

**Figure 6:**
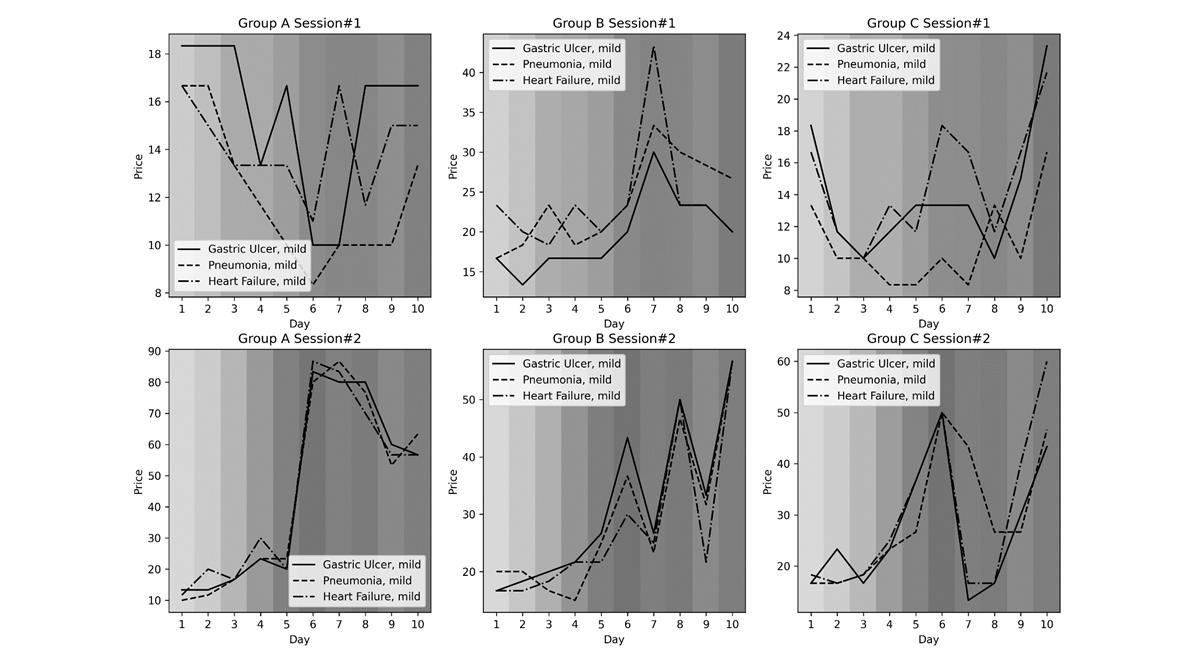
Association between price and workload. The upper graphs show the first session, in which players were asked to collect as many tokens as possible, and the lower graphs show the second session, in which players were asked to make decisions as nurse managers in real practice. A darker gray indicates a higher workload.

When average *workload* increased, participants typically set higher prices for each illness; however, this did not occur every time (Figure 6). Every group, especially Group A, showed a more apparent association between price and workload during the second session than that in first session (Figures S4 and S5 in Multimedia Appendix 1). There was a similar tendency for mild illnesses; however, we could not evaluate quantitative association due to limited data.

## Discussion

### General

A preliminarily examination of practicing nurse managers’ perception on the feasibility of using a token economy–based bed allocation system to mitigate information asymmetry indicated that the system may enhance staff satisfaction and patient safety but may worsen managerial rivalry and inhibit staff development. There is also potential that hospital administrators can utilize our system as a strategy development tool for human resource allocation.

In mathematical modeling studies of bed assignment processes, patient flow, and bed availability [19,20], there is the implicit assumption that there is no information asymmetry, which allows an optimized solution to be found; however, as those studies [19,20] indicated, communication problems between planners and frontline workers remained. That is, information asymmetry diminished the efficacy of such methods in practice. The impossibility of the planner being able to collect complete information has been widely acknowledged in economics (ie, calculation controversy [28]) and by military strategists (fog of war [29]). Likewise, in real practice at hospitals, there is too much apparent or latent information that can be considered in bed assignment process; thus, solving the bed assignment operation problem with mathematical calculation is fundamentally unfeasible.

To the best of our knowledge, no previous research has focused on information asymmetry in bed allocation or hospital management. We identified optimized bed assignment as the target behavior and designed our token economy with transactions occurring between physicians and impatient wards. Unlike token economy designs, in which psychiatrists or teachers define target behavior unilaterally, our design made both stakeholder parties represent desirable behaviors with tokens. Even though the sample size was limited, associations between the information that participants had (*workload*) and the information that participants broadcast (*price*) demonstrated that our token economy–based has potential to mitigate information asymmetry in bed allocation management.

Participants positively rated staff satisfaction, patient safety, and consistency with divisional goals. Autonomy is said to be one of the important factors in improving employee satisfaction in hospitals [30], and characteristics of the proposed method—each frontline division can broadcast their status in a 1-dimensional simplified manner, in the form of pricing—may enhance the autonomy of divisions, resulting in positive effects, which may explain our survey results. Employee satisfaction increases organizational loyalty and employee retention [31], which is critical for human resource management generally and specifically in the nursing filed [32,33]. In addition, patient safety improves when employees are satisfied with their workplace [34] and vice versa [35].

Participants negatively rated the effect on the intensity of managerial rivalry, employee development, and the benefits for patients. Managerial rivalry is said to be a disadvantage of decentralized decision-making;. However, a decentralized method is also said to have positive effects on employee development, by allowing autonomous decision-making [26].

Participants were nurse managers in a teaching hospital, where many employees have limited experience as nurses to make adequate decisions independently; therefore, participants, as managers, might be afraid that their staff would make selfish choices [36]. Given this, prior to being implementing this token economy–based systems in practice, staff decision-making capabilities should be evaluated. Regarding benefit for patients, our method did not take ward specialty into consideration when selecting admission for the sake of simplicity; therefore, participants thought patients had higher possibilities of being assigned to nonspecialized wards than the current method. To address this problem, rule-based restrictions can be considered in future system designs.

When asked about desirable rewards for tokens, most participants indicated human resources (in particular, additional nursing staff). Rewards for tokens are called “back-up reinforcer(s)” [37] when used in behavior modification fields, and are key factors in the method’s design [38]. Hospitals in Japan are suffering from nurse shortages [39], and efficient human resource allocation is needed. Wards (or divisions) that collect more tokens can be regarded as creating more value for the hospital. Therefore, allocating more resources to those wards is reasonable, especially given that hospitals are a labor-intensive industry [40], in which nurses play central role [41]. Through such implementation design, a token economy–based system may be able to optimize resource allocation and improve staff motivation.

Although we chose bed allocation as the target problem, hospitals have many other resource allocation problems to be solved. The proposed token economy–based method may be applied to other resource allocation problems in hospitals in the future, with appropriate token economy design.

### Limitations

The study had some limitations because this research showed only preliminary implications for efficiency in applying a token economy–based system to bed assignment processes. First, the assumptions used in our simulation were too simple compared with those in real practice. That is, we only considered emergency admissions, whereas in reality, hospitals also receive elective admissions. The proposed token economy–based system may cause confusion for nurse managers whether emergency and elective cases should be prioritized at first, but they will be able to adjust pricing behavior by referring elective admission lists. We can, theoretically, expect that the adjustment may result in convergence to adequate allocation via market mechanism. Second, participants were recruited from a single university-affiliated teaching hospital, thus the perceptions of the participants may be different from the perceptions of staff in general hospital fields. Third, we only recruited 9 participants, due to limited resources, and the limited sample size may result in impaired external validity.

Based on these limitations, immediate implementation of our method into real practice is probably unfeasible. However, by showing the constraints of previous studies [19,20] and proposing an innovative method, we make an important contribution.

### Conclusion

A token economy–based bed allocation system has the potential to be an effective method to innovatively solve bed allocation problems in hospitals, by mitigating intraorganizational information asymmetry, and improve staff satisfaction, by allowing the autonomy of frontline professionals.

## Appendix

Multimedia Appendix 1 Supplementary tables and figures.
