# Supplementary material for: Token Economy–Based Hospital Bed Allocation to Mitigate Information Asymmetry: Proof-of-Concept Study Through Simulation Implementation
Source: JMIR Form Res. 2022 Mar 4;6(3):e28877. doi: 10.2196/28877 (PMC8933802; doi:10.2196/28877)
Supplement: Multimedia Appendix 1 [file formative_v6i3e28877_app1.pdf]

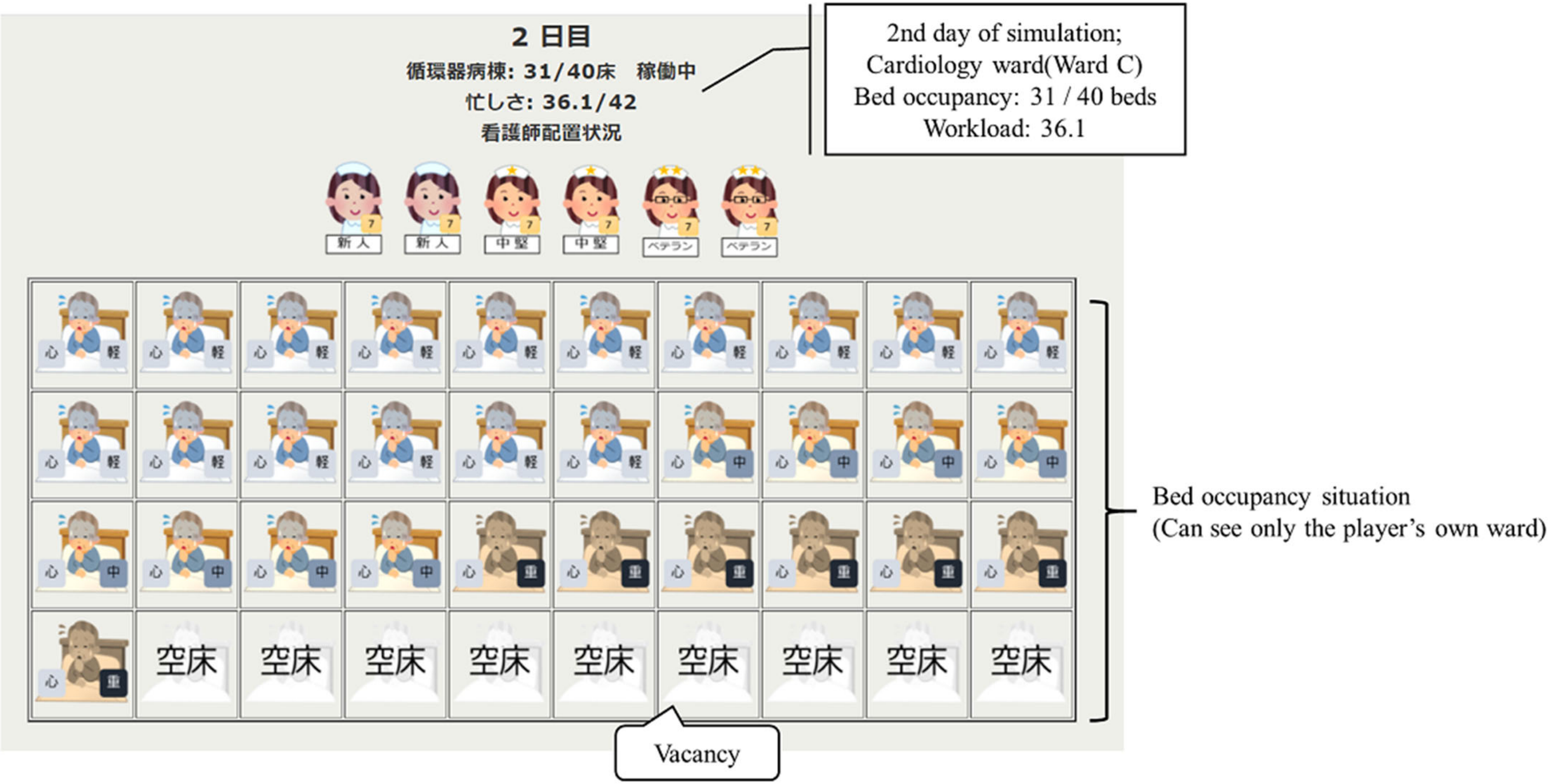

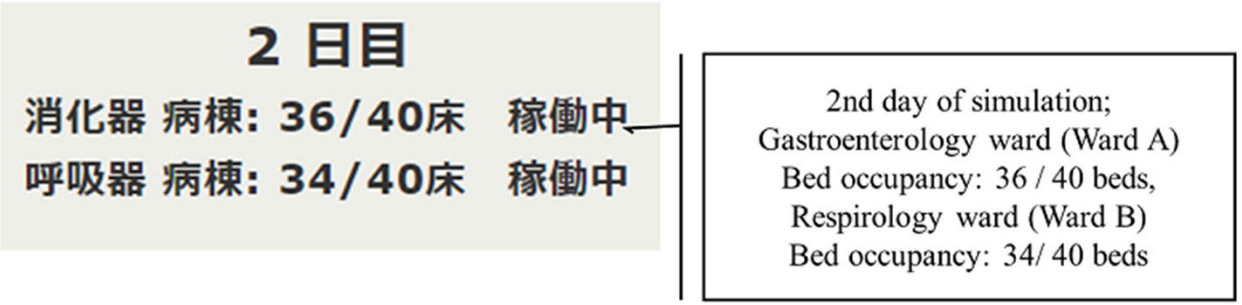

Figure S1:
User interfaces of simulation game showing the data available for players.

Figure S2:
Price inputting interface of simulation game.

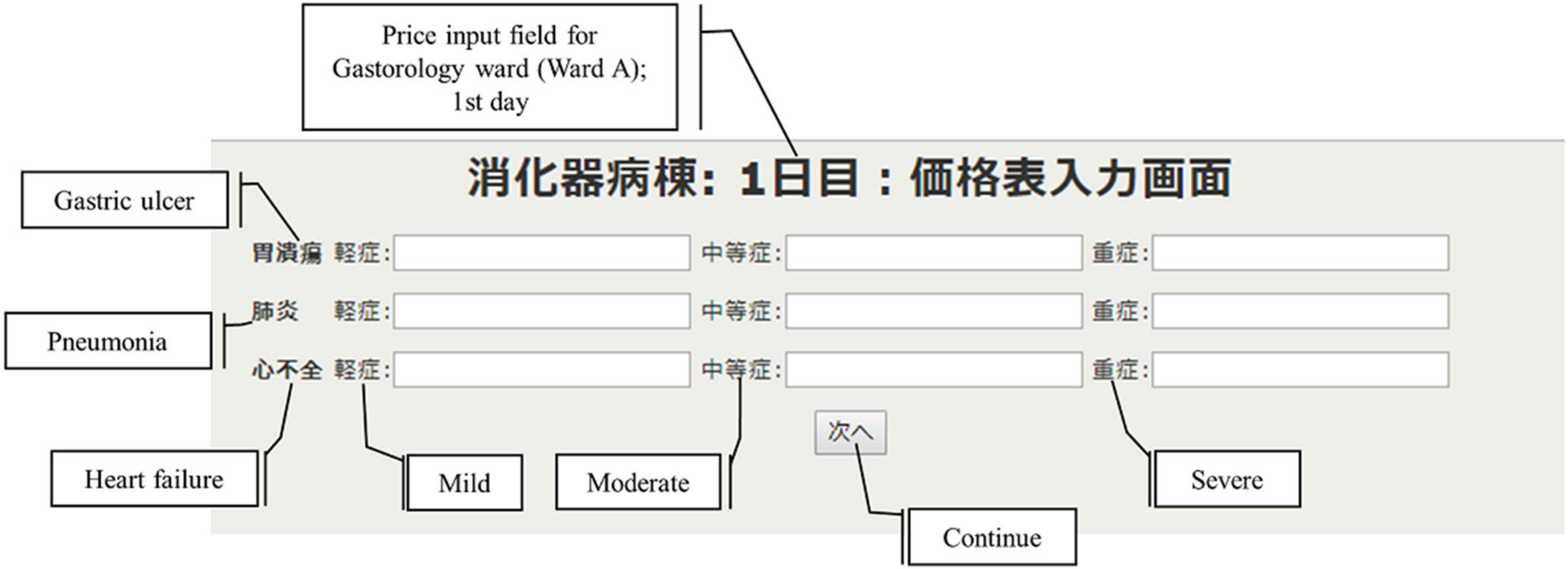

Figure S3:

Information shown to participants when patient arrival, admission, and discharge occurred.

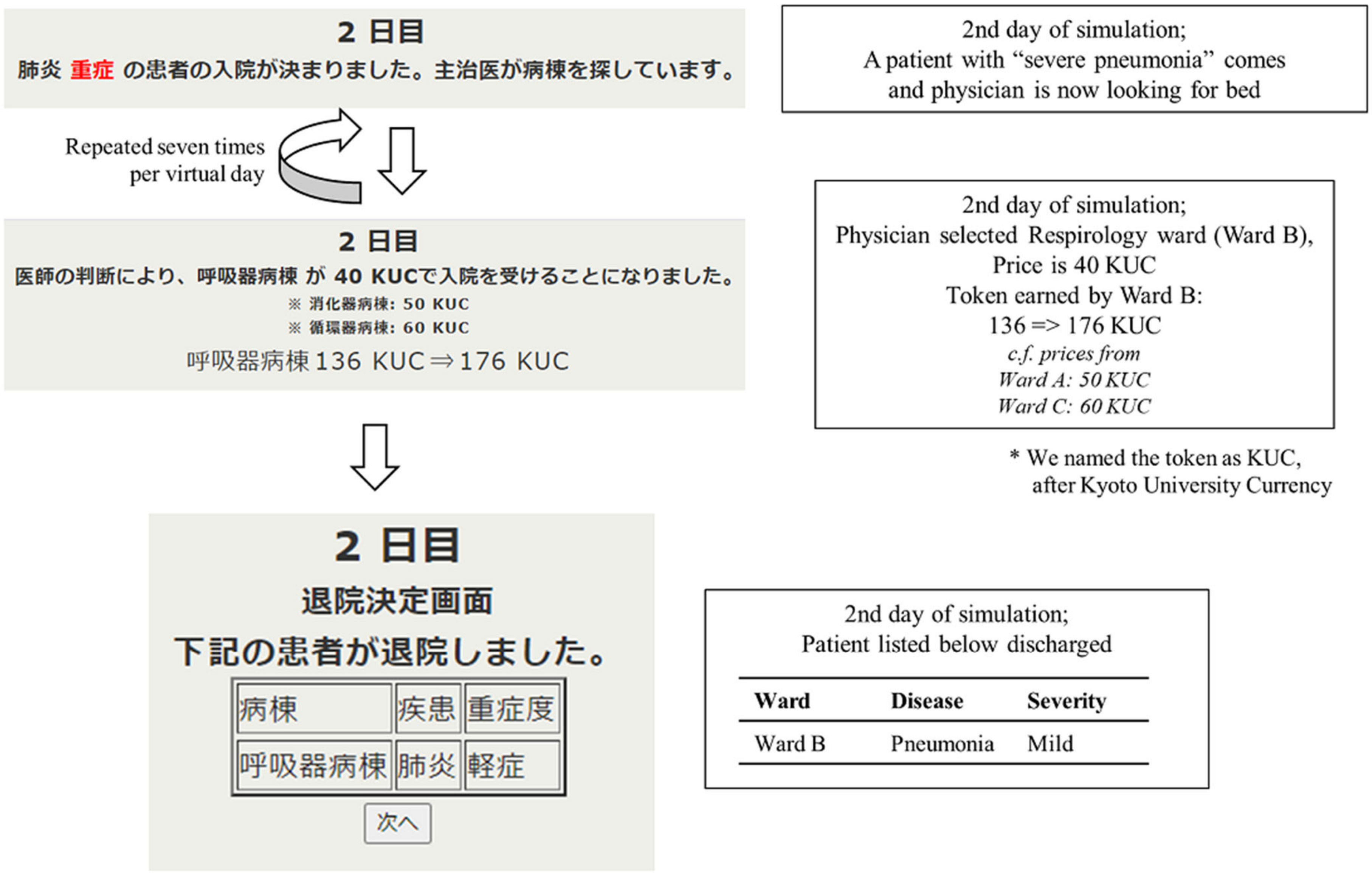

Figure S4:
Association between price fluctuation and workloads in moderate conditions.

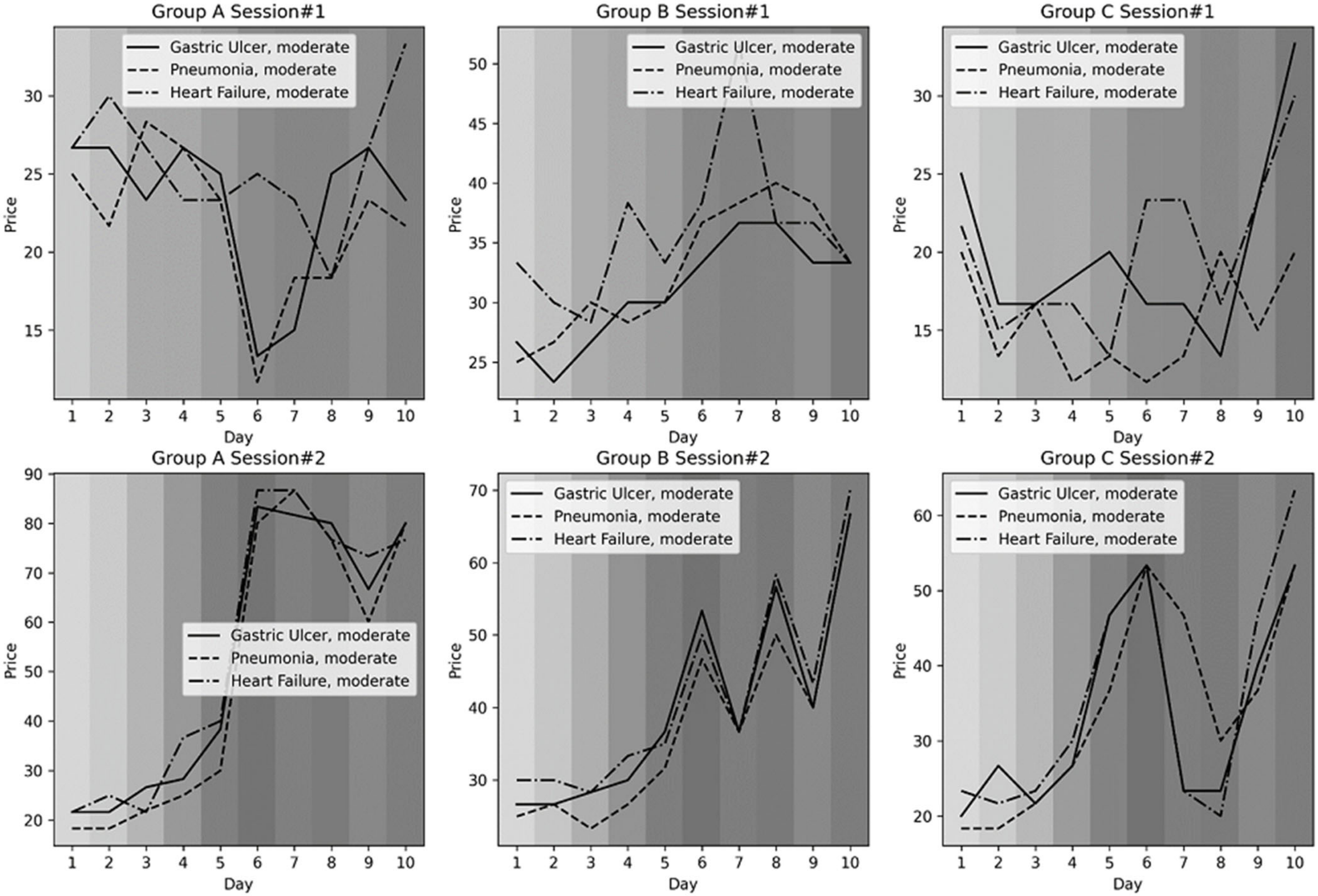

Figure S5:
Association between price fluctuation and workloads in severe conditions.

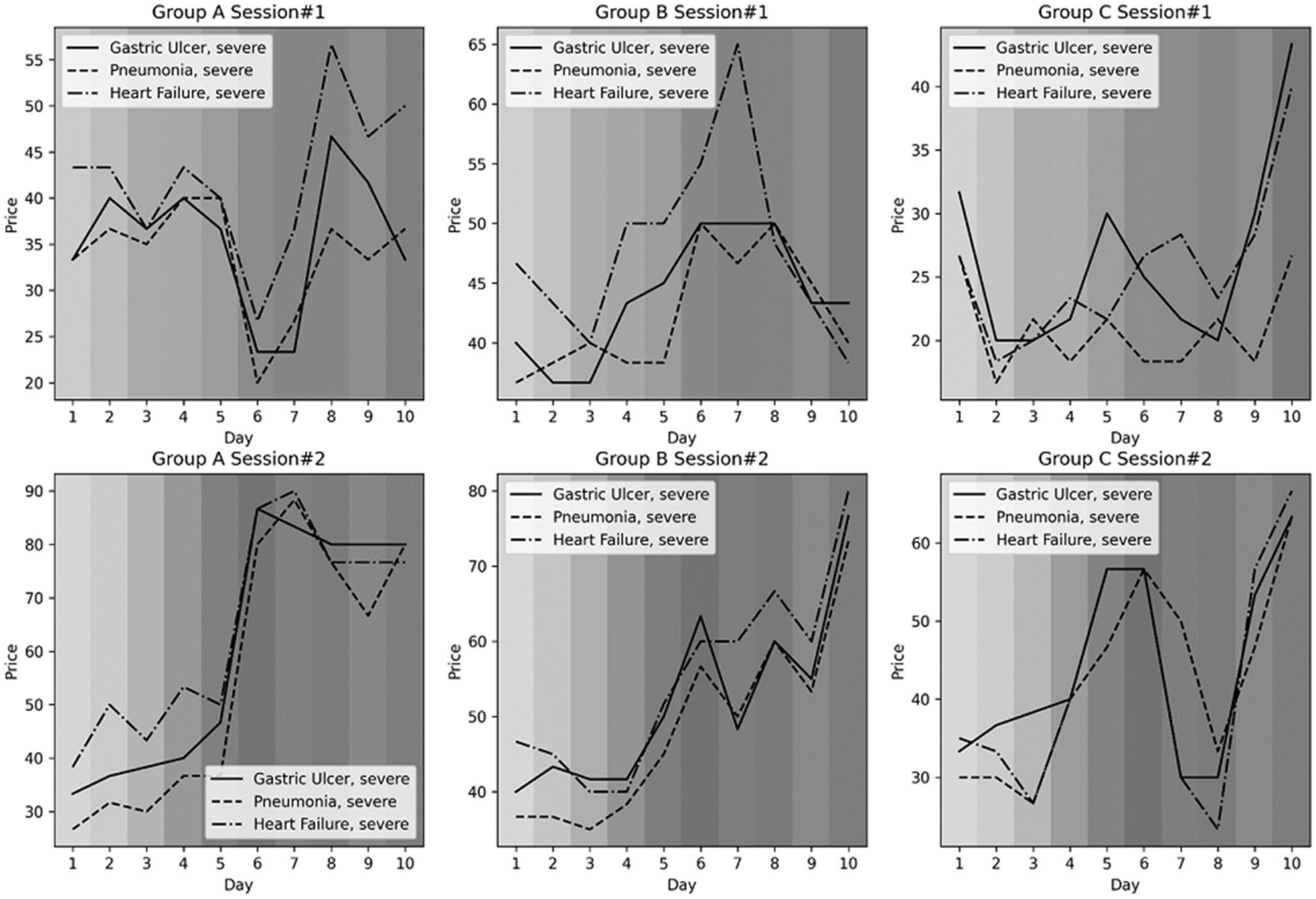

Table S1:
Weighted workload imposed to wards.
Numbers were dependent on whether patients' disease are aligned with wards' specialty and severity of the disease.

Workloads		Disease severity		
		Mild	Moderate	Severe
Specialty	Aligned	1.0	1.2	1.5
	Not aligned	1.5	1.8	2.25
